# Manual Medicine Related Injuries Experienced by Physicians: A Missing Aspect in Therapies Using Manipulation of Joints

**DOI:** 10.1155/2015/507051

**Published:** 2015-02-28

**Authors:** Jost Steinhaeuser, Katja Goetz, Andreas Oser, Stefanie Joos

**Affiliations:** ^1^Department of General Practice and Health Services Research, University Hospital of Heidelberg, Vossstrasse 2, 69115 Heidelberg, Germany; ^2^Institute of Family Medicine, University of Lübeck, Ratzeburger Allee 160, 23538 Lübeck, Germany; ^3^Orthopedic Practice, Wilhelm-Becker-Strasse 11 b, 75179 Pforzheim, Germany; ^4^Institute of General Practice, University Hospital of Tübingen, Österbergstraße 9, 72074 Tübingen, Germany

## Abstract

*Background.* In 2010 Manual Medicine (MM) was the second most common additional qualification among physicians in Germany, which is recommended to be used in several guidelines. Aim of this analysis was to raise the amount of information on MM related injuries (MMri) experienced by physicians at any point of their career while applying MM. *Methods.* Data on MMri of a questionnaire that was used to gain first insights into MM in Germany from a health services research perspective was analysed. *Results.* A total of 301 physicians (20% female) participated in this study. The participants' mean age was 46. 11% of the participants experienced some kind of MMri during their career as a MM provider. In the three worst cases these MMri were fractures and therefore classified as moderate. Mild MMri were joint dysfunction syndromes (*N* = 30), distortions of fingers (*N* = 7), and shoulder pain (*N* = 3). Subgroup analyses showed no significant differences in the rate of MMri when comparing gender, provider organizations for postgraduate MM courses, and medical disciplines. *Conclusion.* Our analysis shows risks for providers of MM. As this analysis suffers from the risk of recall bias, future studies should be performed to get more insights into this aspect of MM.

## 1. Introduction

In Germany, therapies using mobilization or manipulation of joints are called* Manual Medicine* (MM) if provided by a specially trained physician. Until 2003, this additional qualification was named* Chirotherapy*. Nowadays, both titles are used synonymously [1]. This additional qualification can be acquired by physicians after having accomplished postgraduate specialty training and consists of 320 hours of theoretical and practical training. With almost 19.500 physicians in the years 2009/2010, MM was the second most common additional qualification after “emergency medical aid” (28.000 physicians) in Germany [2]. Several German guidelines recommend the use of MM in reasons for encounters of the musculoskeletal system such as neck or low back pain [3, 4].

In previous literature, the discussion about adverse events assumed them to be caused by some kinds of manual manipulation, with the main focus on arterial dissections causing strokes [5–8]. In the past, authors called for further research, employing prospective cohort study designs to uncover both the benefits and the risks associated with cervical manipulative therapy [9]. Grades of adverse events in manual manipulation have been defined by Carnes et al. into major, moderate, and mild [10]. A systematic review performed by the same working group showed that the risk for major adverse events by manual manipulation is about 0.13% and for minor to moderate ones between 22% and 41% [11].

An additional aspect of risks associated with MM is the one experienced by the providers of MM. Experience of the authors performing MM by themselves (Jost Steinhaeuser and Andreas Oser) has shown that physicians can experience some kind of MM related injuries (MMri). However, there is no body of evidence about this aspect of MM in Germany. Therefore, the aim of this analysis was to gather information about MMri in physicians associated with the delivery of MM in Germany.

## 2. Materials and Methods

Data of an online survey, primarily performed to give a first description of relevant health service research aspects of MM in Germany, was secondarily analysed [12]. The 20-item questionnaire used was developed on a selective literature search and personal experiences with MM [13–15]. Whether the items are unambiguous and understandable was tested with two different courses on MM. It was put online between April 2009 and March 2010. The original questionnaire can be found as “Supplementary Material” here: http://link.springer.com/article/10.1007%2Fs00132-011-1750-5.

The analysis presented here focuses on question 10: “Have you ever hurt yourself during performing a MM therapy?” Response options were “no, never before,” “yes, once,” and “yes, several times.” In case “yes” was chosen, it was possible to describe each kind of MMri in an open-ended question.

### 2.1. Recruitment

At the time of the survey, 19.409 physicians had an additional MM qualification in Germany [2]. As there is no central register available of all physicians with the additional qualification or of provider organizations for postgraduate MM courses (POPC), we asked the five POPC known to have the most members to cooperate in the recruitment phase. POPC were asked to invite their members to take part in the study by e-mail and/or by including the link to the survey on their homepage. In addition, participants were recruited by advertisement in a MM journal [16]. One month before closing the survey, the POPC were requested to send a reminder to their members by e-mail.

### 2.2. Statistics

Statistical analyses were performed with SPSS 18.0 (Statistical Package for Social Sciences Inc., Chicago, IL, USA). In case of less than 3% missing values, percentages are given as valid percentages, which means they are summed up to 100%. In the first step, descriptive analysis was performed focusing on frequency, percentages, and cross classified tables. Secondly, an explorative subgroup analysis was performed to explore if MMri are related to gender, POPC, or specialty. Differences between MMri and gender, POPC and specialties were analysed using nonparametric tests (Mann-Whitney *U* test and Kruskal-Wallis test, resp.). A level of significance of *P* ≤ 0.05 was accepted. There was no adjustment for multiple testing performed.

### 2.3. Qualitative Analysis

Rating of MMri: open answers were categorized by Jost Steinhaeuser and Andreas Oser independently. In the next step, categorization was discussed until consensus was achieved. Categorization of MMri as major, moderate, or mild was performed following the definition of adverse events in patients by Carnes et al. [10]. For details, please see Table 1.

### 2.4. Ethical Approval

We involved the ethics committee of Heidelberg University in the context of a preceding study (also a survey among physicians). In this context, they informed us that an ethical approval is not necessary in survey studies among physicians raising data anonymously. Therefore, we did not involve the ethics committee again. An introductory text within the consent form informed participants of the voluntary and anonymous character of the survey.

## 3. Results

301 physicians from all over Germany participated in the survey of which 296 completed the questionnaire.  Table 2 shows the demographic data of participating physicians. Participants represent about 1.5% of all physicians in Germany with the additional qualification MM at that time [2] (see Table 2).

### 3.1. MMri Experienced by Physicians

During their career, thirty-three physicians (11%) experienced some kind of MMri. Of these participants, 17 (52%) experienced MMri more than once. Three participants gave no further description of what kind of MMri they experienced. No major MMri were reported in accordance with the defined grades for adverse events in patients. Moderate MMri occurred in three cases as fractures. Mild MMri were experienced in 30 cases of joint dysfunction syndromes (meaning a physiological barrier limiting range of movement) of different areas of the spine, distortions of fingers (*N* = 7), and shoulder pain (*N* = 3). One participant got slapped in the face by a frightened patient. Furthermore, there were three MMri assumed by the participants which could not be classified in the grading system (degenerative cervical spine, carpal tunnel syndrome, and inguinal hernia) (see Table 3).

### 3.2. Subgroup Analyses

Subgroup analyses comparing gender, POPC, and specialties showed no significant differences in MMri.

## 4. Discussion

To our knowledge, this is the first analysis gathering data on MMri in MM among physicians. Of the 301 mainly male physicians from Germany participating in this study, forty-four distinct MMri have been reported. These MMri were mainly mild with the exception of three cases of fractures. The grading system used by Carnes et al. to report adverse event in patients has shown to be applicable for grading MMri in physicians.

MM therapy involves some risk to experience MMri for providers. The finding that subgroup analyses showed no significant differences regarding gender, different POPC and disciplines, and self-experienced MMri might indicate that there are no major differences between the risks of experiencing MMri within different MM techniques thought by different POPC. Future studies should address this hypothesis. These future studies could, for example, collect data on the explicit circumstances in which the MMri occurred.

From a health care system perspective, there is literature pointing in the direction of MM care being a relatively cost-effective treatment of common and high prevalent diseases such as lower back pain and neck pain [17]. From the patient's perspective, there is evidence that MM is safer than the use of NSAIDs [18]. A former future study showed that physicians express a wide range of mostly positive views and experiences when applying MM in Germany [19]. Additionally we know physicians having a higher job satisfaction if they have the opportunity to use their abilities [20]. In this context, MM is a welcome change for physicians, who perceived the routine care of their profession as rather distant from the patient [20]. MM can therefore enhance job satisfaction. Future research should include more aspects of physicians' well-being as it is quite likely also influencing the delivery of MM [21].

### 4.1. Strength and Limitations

The strength of this study is that for the first time data about MMri in MM among physicians is available from different POPC in Germany. However, as the study was originally performed for a different reason, we did neither ask participants which exact technique they used nor ask them the exact type of complaint of patients treated. The questionnaire used was not formally validated. Nevertheless, it was pilot-tested by 22 physicians. A general limitation of an online survey is that there is no possibility to calculate a response rate, because the number of reached participants is not known. However, the distribution between the sexes and age of the participants was comparable to the total sample of MM physicians in Germany. In 2009/2010 about 19% of female physicians had an additional MM qualification and the average age was between 41 in physicians working in a hospital and 52 in physicians working in the ambulatory sector [2, 22]. Furthermore, selection bias, recall bias, life time bias, and underreporting might be serious limitations of our analysis. We do not know, for example, if nonresponders are clinicians who experienced more MMri than responders. Therefore, future research should comprise these aspects.

## 5. Conclusion

Our study shows that MM therapy involves risks of experiencing an injury for providers. The grading system used for patients' adverse events can be transferred to grade physicians' MMri, too. Most serious MMri were fractures. More specific data about the incidence and context of MMri involving different MM techniques should be monitored.

## Figures and Tables

**Table 1:** Definition of grades of adverse events in patients [10].

Grade of adverse event	Definition
Major adverse event (e.g., dissection of a cervical artery)	(i) Medium to long term
	(ii) Moderate to severe intensity
	(iii) Further treatment required

Moderate adverse event (e.g., rib fracture)	(i) Medium to long term
	(ii) Moderate in severity

Mild adverse event (e.g., dizziness)	(i) Short term
	(ii) Moderate intensity

**Table 2:** Study sample.

	*N*	%
Sex		
Female	60	(20)
Male	241	(80)
Age, medium (min–max)	46 (28–72)	
Working in a hospital	51	(17)
Practice form		
Single handed practice	104	(34)
Group practice	142	(47)
Location		
Country	126	(42)
City	170	(56)
Specialty		
Family medicine	142	(47)
Orthopaedic surgeon	65	(22)
Others	68	(24)
Residents	21	(7)

**Table 3:** Type and number of Manual Medicine related injuries experienced by physicians.

Grades of Manual Medicine related injuries	Classification of Manual Medicine related injuries	Affected part of the body	Number
Major	None		

Moderate	Fracture	Of a carpal bone	(*n* = 1)
		Of a rib	(*n* = 2)

Mild	Joint dysfunction syndrome (physiological barrier limiting range of movement)	Spine, not specified	(*n* = 8)
		Sciatic pain	(*n* = 8)
		Thoracic spine	(*n* = 7)
		Lumbar spine	(*n* = 6)
		Cervical spine	(*n* = 1)
	Distortion	Finger, not specified	(*n* = 3)
	Pain	Thumb	(*n* = 3)
		Digitus index	(*n* = 1)
		Shoulder	(*n* = 3)
	Slap in the face		(*n* = 1)

Others		Inguinal hernia	(*n* = 1)
		Cervical spine degeneration	(*n* = 1)
		Carpal tunnel syndrome	(*n* = 1)
		No further description	(*n* = 3)
